# Immunomodulation by Mesenchymal Stem Cells (MSCs): Mechanisms of Action of Living, Apoptotic, and Dead MSCs

**DOI:** 10.3389/fimmu.2019.01191

**Published:** 2019-06-04

**Authors:** Andreas Robert Rudolf Weiss, Marc Hendrik Dahlke

**Affiliations:** ^1^Department of Surgical Sciences, Dunedin School of Medicine, University of Otago, Dunedin, New Zealand; ^2^Department of Surgery, Robert Bosch Hospital, Stuttgart, Germany

**Keywords:** mesenchymal stem cell (MSC), immunomodulation, immunogenicity, tumor induction, apoptosis, HI-MSC, monocytes, regulatory T cells

## Abstract

Expectations on mesenchymal stem cell (MSC) treatment are high, especially in the fields of sepsis, transplant medicine, and autoimmune diseases. Various pre-clinical studies have been conducted with encouraging results, although the mechanisms of action behind the observed immunomodulatory capacity of mesenchymal stem cells have not been fully understood. Previous studies have demonstrated that the immunomodulatory effect of MSCs is communicated via MSC-secreted cytokines and has been proven to rely on the local microenvironment as some of the observed effects depend on a pre-treatment of MSCs with inflammatory cytokines. Nonetheless, recent findings indicate that the cytokine-mediated effects are only one part of the equation as apoptotic, metabolically inactivated, or even fragmented MSCs have been shown to possess an immunomodulatory potential as well. Both cytokine-dependent and cytokine-independent mechanisms suggest a key role for regulatory T cells and monocytes in the overall pattern, but the principle as to why viable and non-viable MSCs have similar immunomodulatory capacities remains elusive. Here we review the current knowledge on cellular and molecular mechanisms involved in MSC-mediated immunomodulation and focus on the viability of MSCs, as there is still uncertainty concerning the tumorigenic potential of living MSCs.

## Introduction

Mesenchymal stem cell (MSC) therapy offers a promising treatment option for autoimmune diseases, sepsis, and in transplant surgery (1–7). However, the underlying cellular and molecular mechanisms of MSC-mediated immunomodulation have not been fully clarified. Studies have demonstrated various immunomodulatory changes following the administration of MSCs, although a clear picture is still missing and study results are often inconsistent. This might partially be explained by the fact that MSCs from different sources and under different culture conditions express different surface markers, show varying cytokine secretion profiles and differ in telomere-length and methylation patterns (8–15).

However, comparing the available data is complicated by a lack of standardization for the isolation, culture, and characterization of MSCs (16). MSCs can be harvested from various adult tissues such as bone marrow, adipose tissue, inner organs, and peripheral blood as well as from neonatal tissues (e.g., umbilical cord, placenta, amniotic fluid, amniotic membrane). In clinical studies, adipose tissue, and umbilical cord-derived MSCs have regularly been used due to their accessibility. The broad range of potential sources makes a comparison of study results challenging, as MSCs display varying features *in vitro* and *in vivo* depending on the tissue they originate from (17–19). In most study protocols MSCs were administered intravenously, yet in others they were delivered via an intraarterial, intraportal, intraperitoneal, or topical route or were administered directly into the damaged tissue (20–24).

Furthermore, freshly thawed MSCs seem to have an impaired immunomodulatory capacity compared to continuously cultured MSCs (25). The fact that MSCs act differently depending on the local microenvironment contributes even more to the complexity of understanding MSC-mediated immunomodulation (26–28). MSCs have a short half-life and cannot pass through the lung capillary network after IV administration, which appears to contradict the observed long-term immunomodulatory effects, particularly in transplant settings (29, 30).

Nevertheless, there are certain patterns and pathways that seem to be consistent and have been repeatedly demonstrated. MSC-mediated immunomodulation operates through a synergy of cell contact-dependent mechanisms and soluble factors (8, 31). MSCs reveal their immunomodulatory potential via functional changes of monocytes/macrophages, dendritic cells, T cells, B cells, and natural killer cells (6, 27, 32–36). In particular, anti-inflammatory monocytes/macrophages and regulatory T cells (Tregs) play a prominent role as they unfold their full immunomodulatory potential in a complex interaction catalyzed by MSCs (32, 37, 38). The interaction between MSCs, monocytes, and Tregs have often been attributed to MSC-secreted cytokines, although there is increasing evidence for mechanisms that rely on a direct cell-cell interaction, which—in the case of MSCs—does not necessarily require an intact cell metabolism (27, 31, 39, 40). Recent studies could demonstrate that apoptotic, metabolically inactivated, or even fragmented MSCs possess immunomodulatory capacities (21, 39, 41). As there are still ongoing concerns as to what extent living MSCs might contribute to tumorigenesis, the option to use dead cells or even cell fragments could be a promising alternative. This review summarizes the current knowledge on cellular and molecular interactions in MSC-derived immunomodulation by highlighting the different immune responses to living, apoptotic, and dead MSCs and provides an overview of the potential risks of MSC treatment in terms of tumor induction.

## Immunomodulation by Living MSCs

### Effect on Monocytes/Macrophages and Dendritic Cells

**Figure 1:**
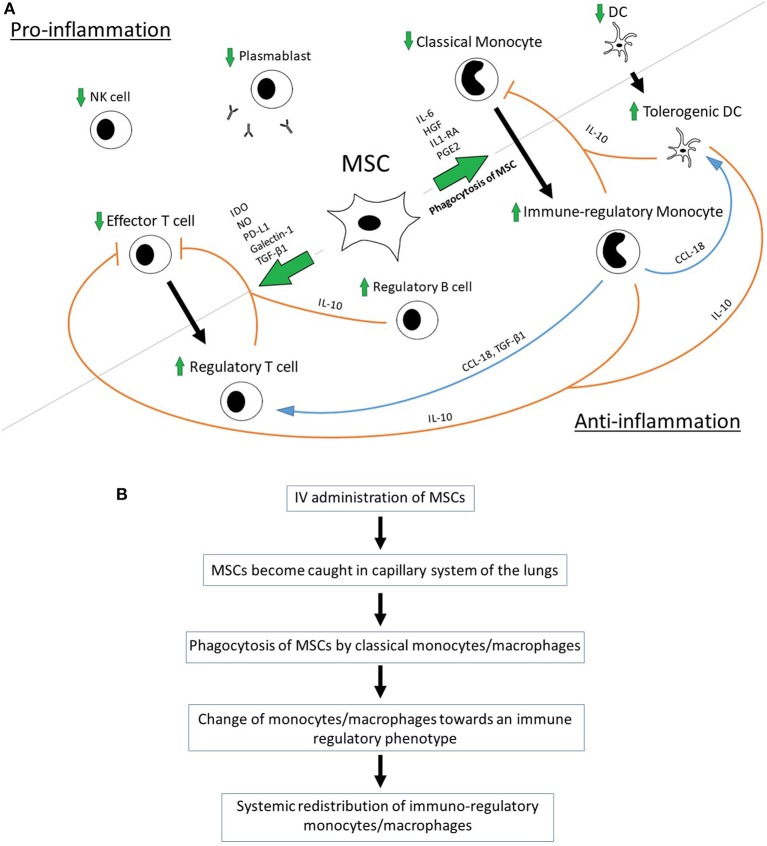
**(A)** Proposed interaction of MSCs with host immune cells. **(B)** Proposed pathway of MSC-mediated anti-inflammation via phagocytosis of MSCs [in accordance with De Witte et al. (31) and Braza et al. (53)].

MSC were shown to promote the polarization of monocytes/macrophages toward an anti-inflammatory/immune-regulatory (type 2) phenotype and to directly inhibit the differentiation into the type 1 phenotype and dendritic cells (DCs) (10, 42–45). MSC-secreted Interleukin 1 Receptor Antagonist (IL1-RA) can promote the polarization of macrophages toward the type 2 phenotype (36). Anti-inflammatory monocytes secret high levels of IL-10 and have decreased levels of IL-12p70, TNF-a, and IL-17 expression—a process that is mediated by MSC-produced IL-6 and hepatocyte growth factor (HGF) (10, 40). A key role for the MSC-mediated, increased production of IL-10 has been demonstrated in a sepsis model in mice where IL-10 neutralization reversed the beneficial effect of bone marrow-derived MSCs on overall survival after induction of sepsis via cecal ligation and puncture (CLP) (6). Monocyte-derived IL-10 prevents monocyte differentiation into DCs and shifts monocytes toward an anti-inflammatory, IL-10-secreting subtype in terms of a positive-feedback loop (10). Apart from IL-10, MCS-primed monocytes express high levels of MHC class II, CD45R, and CD11b and seem to be able to suppress T-cell activity regardless of FoxP3^+^ Tregs (46). The supernatants of type 2 macrophages induce the formation of FoxP3^+^ Tregs from naïve CD4^+^ T cells, which emphasizes the role of soluble factors in MSC-mediated immunomodulation (47). The monocyte-induced Treg-formation is mediated by monocyte-produced CCL-18 and monocyte-released transforming growth factor beta 1 (TGF- β1) (45, 47). Macrophages bind and re-release TGF-β1 during their differentiation into type 2 macrophages and might thereby contribute to the MSC-induced formation of Tregs as MSCs have been shown to secrete TGF-β1 (45, 47). The neutralization of CCL-18 leads to a significant reduction in MSC-induced Treg formation (45). CCL-18 can turn memory CD4^+^ T cells into to CD4^+^CD25^+^Foxp3^+^ Tregs with an increased IL-10 and TGF- β1 production. CCL-18-pretreated Tregs inhibit CD4^+^CD25^−^ effector T cell proliferation via the activation of G-protein-coupled receptors (48). Macrophage type 2-derived CCL-18 can differentiate DCs into tolerogenic DCs, which are in turn able to prime Tregs (45, 48, 49) (Figure 1A). Interestingly, high concentrations of CCL-18 producing antigen-presenting cells can be found in the lungs, where MSCs become caught in the capillary system after IV application (50–52) (Figure 1B).

MSCs also suppress the migration and maturation of DCs (32). In the presence of MSCs, DCs are less capable to support antigen specific CD4^+^ T cell proliferation and to display an MHC class II-peptide complex (54). Mature type 1 DCs secrete significantly less TNF-α, if co-cultured with MSCs and anti-inflammatory mature type 2 DCs show an increased IL-10 secretion (55). Furthermore, Sca-1^+^CD117^−^Lin^−^ bone marrow-derived MSCs have been shown to generate regulatory DCs with immune regulatory function from hematopoietic stem cells in mice (56).

Recently, a cytokine-independent pathway for the MSC-induced polarization of monocytes/macrophages has been revealed. In a mouse model of asthma, MSCs were phagocytosed by lung macrophages. The phagocytosis of MSCs caused monocytes to turn into a type 2 immunosuppressive phenotype (53). A previous study from 2012 observed a similar pattern in a p. aeruginosa peritonitis model in mice (57). The intravenous application of bone marrow-derived MSCs was followed by an increased phagocytic activity in blood monocytes compared with the PBS control group. Furthermore, an increase in alternately activated CD163^+^CD206^+^ monocytes/macrophages in the spleen of the MSC-treated mice could be observed (57). De Witte et al. (31) could show that phagocytosed MSCs are mainly found in non-classical Ly6C^low^ monocytes. The phagocytosis of MSCs caused CD14^++^CD16^−^ classical monocytes to polarize toward a CD14^++^CD16^+^CD206^+^ immune regulatory intermediate subtype with anti-inflammatory properties and increased expression of IL-10 and programmed death ligand-1 (PD-L1). After phagocytosis of MSCs, these primed monocytes were able to induce CD4^+^CD25^hi^ Treg formation *in vitro* to a significantly higher extent than un-primed monocytes (31). Likewise, an increase in anti-inflammatory Ly6C^low^ monocytes in the blood, heart, and spleen was observed after IV administration of MSCs in a Coxsackievirus B3-induced myocarditis model in mice (58).

The depletion of phagocytic cells demonstrates their indispensable role in MSC-mediated immunomodulation as the absence of monocytes/macrophages and dendritic cells abrogates the ability of MSCs to suppress T cell proliferation *in vitro* and their immunomodulatory effect in *in vivo* transplant models (59).

### Effect on T Cells

MSCs suppress T cell proliferation (CD4^+^ and CD8^+^ T cell subsets) in mixed lymphocyte reactions in a dose-dependent manner (39, 60, 61). In interaction with DCs, MSCs cause a shift from pro-inflammatory Th1 to anti-inflammatory Th2 cells including a change in the cytokine profile toward anti-inflammation (32, 62–64). Moreover, MSCs facilitate the formation of Tregs *in vitro* and *in vivo* (3, 32, 55). Tregs are essential for the immune homeostasis by preventing autoimmunity (65, 66). The induction of CD4^+^CD25^+^FoxP3^+^ Tregs is a mainstay in MSC-mediated immunomodulation and was shown to be essential for tolerance induction in a kidney allograft transplantation model (32). The MSC-induced upregulation of Tregs does not result from an expansion of pre-existing natural Tregs but via induction of Tregs from conventional T cells (67, 68). TGF-β1-neutralization studies have shown that the generation of Tregs is TGF-β1-mediated and that MSCs constitutively secrete TGF-β1. However, TGF-β1 seems not to be sufficient on its own, as the presence of monocytes was proven to be essential for the formation of Tregs (45). In co-treatment with MMF, MSCs have been shown to promote a direct conversion of IL-17A^+^ cells into IL-17A^neg^Foxp3^+^ Tregs (35). MSCs also constitutively secrete indoleamine 2,3-dioxygenase (IDO) and the secretion is increased upon stimulation by INF-γ. The consecutive tryptophan depletion leads to an inhibition of allogeneic T cell responses, stimulates the secretion of IL-4 in Th2 cells and decreases the IFN-γ production by Th1 cells (27, 32, 55, 69, 70). Gieseke et al. have shown that MSCs can directly inhibit the proliferation of alloreactive CD4^+^ and CD8^+^ T cells without the presence of other immune cells and that this process is partially mediated by MSC-derived galectin-1 (60). Via the secretion of PD-L1, MSCs can suppress T cell activation and induce an irreversible T cell hypo-responsiveness and apoptosis (71, 72) (Figure 1A).

### Effect on B Cells

MSCs directly interact with B cells and are able to reduce plasmablast formation as well as to promote the induction of regulatory B cells (Bregs) (73). Bregs have immunosuppressive properties through which they provide immunological tolerance (74). IL-10-producing Bregs were shown to convert effector CD4^+^ T cells into Foxp3^+^ Tregs (75). The stimulatory effect of MSCs on Breg formation and IL-10 production is not mediated via soluble factors but seems to be dependent on direct cell-cell contact or at least on a close proximity of the corresponding cells (27). However, it was shown that the stimulatory effect of MSCs on Breg formation and their suppressive effect on T cell proliferation requires an active cell metabolism (27, 41). Luz-Crawford et al. revealed a cytokine-triggered mechanism, by which MSC-secreted IL1-RA inhibits B cell differentiation (36). In the presence of T cells, MSCs also inhibit the proliferation of B cells, which could be due to T cell-secreted IFN-γ as IFN-γ pre-treated MSCs are able to inhibit B cell proliferation (27).

### Effect on Natural Killer Cells

MSC are also strong inhibitors of natural killer cell (NK cell) proliferation. NK cells show an impaired cytotoxic activity and cytokine production after co-culture with MSCs. There is evidence that the inhibitory effect of MSCs on NK cells involves MSC-secreted prostaglandin E2 (PGE2), IDO, TGF-β1, IL-6 and nitric oxide (NO) (10, 33, 76) (Figure 1A).

## Immunomodulation by Apoptotic and Dead MSCs

### Effect of Apoptotic MSCs

The viability of MSCs does not appear to be a prerequisite for some of their immunomodulatory effects. Apoptotic adipose tissue-derived MSCs (A-ADMSCs) have been shown to reduce mortality in rats after sepsis induction via CLP (21) (Table 1). Mortality, circulating TNF-α level as well as circulating and splenic levels of T helper cells and cytotoxic T cells following CLP were significantly lower in the group treated with A-ADMSCs compared to CLP alone (21). A study group around Chen et al. provided similar results in a CLP-induced model of acute kidney injury in mice with a reduced splenic level of T helper cells and cytotoxic T cells as well as a lower circulating TNF-α level in the group treated with intravenous A-ADMSCs compared to the CLP control group (77). Interestingly, the study by Chang et al. could not prove any benefit and even a trend toward a reduced survival after administration of living MSCs (21). In keeping with the results of Chang et al. it was demonstrated that an IV A-ADMSC treatment is superior to a treatment with living MSCs in a CLP-induced sepsis model in rats (78). The parameters for sepsis-induced acute lung injury (ALI) and acute kidney injury were significantly lower in the group treated with apoptotic MSCs. Furthermore, A-ADMSC treatment was more effective in reducing inflammation, oxidative stress, and apoptosis as well as sepsis-induced histopathological alterations in the lungs and kidneys compared to living MSCs (78). Likewise, A-ADMSCs were shown to be superior to living MSCs for the treatment of an acute lung ischemia-reperfusion injury in rats if administered along with melatonin (79).

**Table 1:** Overview of important immunomodulatory effects of living, apoptotic, and dead MSCs.

	**Living MSCs**	**Apoptotic MSCs**	Dead MSCs^*^
Inhibition of T cell proliferation in MLR	+	n.a.	–
Induction of regulatory T cells *in vivo*	+	n.a.	n.a.
Modulation of monocyte function *in vivo*	+	(+)^**^	+
Attenuation of sepsis *in vivo*	+	+	+

* *Heat-inactivated MSCs without active cell metabolism according to Luk et al. (41)*.

** *Indirect evidence in study from Galleu et al. (80)*.

*n.a. information not available*.

However, there are several studies that demonstrated a significant positive effect of living MSCs in the attenuation of sepsis in different animal models (22, 81–83). Interestingly, a recent study demonstrated that recipient cytotoxic cells cause perforin-induced apoptosis in infused MSCs (80). The apoptosis of MSCs was the prerequisite for MSCs to unfold their immune-regulatory effect in a murine graft vs. host model. Hence, the cytotoxic activity against MSCs was demonstrated to be a crucial part in MSC-mediated immunomodulation. The recognition by cytotoxic cells in this model was shown to be MHC-independent and non–antigen-specific. Moreover, the use of apoptotic MSCs skipped the need for recipient cytotoxic cells. Apoptotic MSCs were immunosuppressive in a TH2-type inflammation model and induced the IDO production in recipient phagocytes (80) (Table 1). Another study provided evidence that the supernatants of macrophages that phagocytized apoptotic mesenchymal stem cells improve the survival of hypoxic cardiomyocytes (84). These findings are in keeping with the “dying stem cell hypothesis” published by Thum in 2005, which stated that the apoptosis of MSCs causes a modulation of the local immune response with a down-regulation of the innate and adaptive immunity (85).

Contrary findings were provided in an endotoxin-induced ALI model in mice. The intrapulmonary administration of apoptotic MSCs did not improve survival or decrease the severity of an endotoxin-induced ALI. Moreover, no decrease in TNF-α levels and no increase in IL-10 levels could be observed, neither in plasma nor in fluid from bronchoalveolar lavage (22). Compared with the above mentioned studies, the intrapulmonary application via trachea/bronchi was a unique characteristic of this study compared to the commonly used IV route and might explain the differing results.

### Effect of Metabolically Inactivated MSCs (HI-MSCs) and MSC Cell Fragments

In 2016, a heat-inactivation protocol for MSCs was introduced, in which human MSCs were heated for 30 min to 50°C (41). Heat-inactivation causes an irreversible cessation of the metabolic and proliferative activity of MSCs. HI-MSCs do not secrete cytokines but their cell integrity remains largely intact. Over the course of time, HI-MSCs are subject to physical disintegration rather than to apoptosis, as they do not overexpress heat shock proteins Hsp27 and Hsp70 and pro-apoptotic Bax (41). Therefore, in this review heat-inactivated MSCs (HI-MSCs) are referred to as being “dead.” In contrast to living MSCs, HI-MSCs do not inhibit T cell proliferation and do not induce Breg formation in mixed lymphocyte reactions. However, HI-MSCs are still able to attenuate the inflammatory response in mice following LPS-administration. After administration of HI-MSCs, serum levels of IFN-γ were significantly reduced whereas IL-10 serum levels were increased (41). MSCs and HI-MSCs show similar effects on monocyte function with significantly reduced production of TNF-α in response to lipopolysaccharide (41). Even MSC-derived membrane particles seem to possess immunomodulatory properties. Goncalves et al. used MSC-derived membrane particles with a size ranging between 63 and 700 nm (>95% smaller than 200 nm). These MSC-derived membrane particles were shown to be enzymatically active but did not suppress T cell proliferation in mixed lymphocyte reactions. The MSC membrane particles were taken up by monocytes and became bound to their plasma membranes thus inducing selective apoptosis of pro-inflammatory CD14^+^CD16^+^ monocytes (39) (Table 1).

## Scene of the Event

There is an ongoing discussion, as to whether MSCs are able to migrate to the site of inflammation/tissue damage or to a transplanted organ. In that context, it is worthwhile to differentiate between endogenous MSCs and exogenously administered MSCs. The data concerning the migratory capacity of endogenous MSCs is controversial and there is no convincing evidence that endogenous MSCs find their way to the site of inflammation/tissue damage via the bloodstream (86). Nevertheless, endogenous MSCs seem to be able to migrate within the tissues and might thereby reach the damaged or inflamed areas (50). A recently published study revealed that exogenous MSCs, if transplanted directly into the tissue, could survive up to 4 months at the effector site with few of these transplanted MSCs beginning to develop a resident cell tissue phenotype (23).

However, in most clinical and pre-clinical models MSCs were administered intravenously. Interestingly, the majority of IV-administered MSCs does not pass the capillary system of the lungs, which contradicts the suggestion that MSCs exert their immunomodulatory effect by migrating to the sites of harm. Within 24 h after being trapped in the pulmonary system a major decrease in the number of viable MSCs can be observed (29, 30). The fate of these MSCs was further unveiled by demonstrating that MSCs become phagocytosed by blood-derived monocytes and neutrophils after their initial entrapment in the lungs and thereby become redistributed systemically with a major accumulation in the liver and the spleen (31, 87) (Figure 1B).

The change in the monocytes' profile toward an immuno-regulatory phenotype after phagocytosis of MSCs followed by their systemic redistribution might be the key mechanism to explain the contradictory findings with regards to the short half-life of MSCs and the long lasting immunomodulatory effects that have been observed (31, 35).

It remains unclear, whether living MSCs are subject to phagocytosis by host-innate immune cells or if MSCs have to undergo apoptosis in order to become phagocytosed. Galleu et al. have shown that infused living MSCs are subject to perforin-induced apoptosis through recipient cytotoxic cells (80). These findings emphasize the importance of apoptosis in MSC-mediated immunomodulation and might explain previous study results, in which apoptotic MSCs were shown to be superior compared to living MSCs (21, 78, 79). Nevertheless, apoptosis cannot explain by which means dead HI-MSCs—which are subject to physical disintegration rather than to apoptosis—unfold their immunomodulatory potential (27, 41).

## Autologous vs. Allogenic—Evidence for Immunogenicity of MSCs

Allogeneic MSCs are frequently used in clinical studies even though their immunogenic potential has not always been taken into account (88). In the past, MSCs were thought to be immune privileged. Several pre-clinical studies could show that both, autologous and allogenic MSCs suppress T cell proliferation in mixed lymphocyte reactions in a dose-dependent manner (61, 89). In animal models, donor-derived MSCs prolonged the survival of semi-allogeneic heart transplants in mice via the generation of Tregs and a shift in the Th1/Th2 balance toward Th2 (90, 91). Interestingly, the IV administration of MSCs is followed by a systemic inflammatory response with an increase in macrophages in lung tissue 2 h after infusion and increased serum levels of pro-inflammatory IL-6, CXCL1, and monocyte chemoattractant protein-1. The phase of acute inflammatory response seems to be followed by a phase with reduced immune reactivity, which might partially explain the increased allograft survival in animal models, in which MSCs were administered prior to transplantation (34, 59, 92).

However, donor-derived MSCs given prior to transplantation only prolonged the survival of allogenic heart grafts if a short course of mycophenolate mofetil (MMF) was administered from the day of transplantation onwards. The administration of donor-derived MSCs alone caused a prompt T cell infiltration of the grafts with consecutive graft rejection, which supports the assumption that allogeneic MSCs are immunogenic and sensitize the recipient (34, 59). It has been demonstrated that allogenic MSCs can activate T cells (89, 93). The additional treatment with MMF seems to eliminate these activated T cells with a consecutive reduction of T cell infiltration in the respective allografts (59).

Further evidence that MSCs are not immune privileged has been provided in a study, in which syngeneic, erythropoietin-releasing MSCs persisted for more than 200 days, whereas allogeneic MSCs were rapidly rejected (94). Zangi et al. provided similar findings with luciferase-labeled MSCs. The survival of allogeneic MSCs was significantly shorter compared to syngeneic MSCs. Moreover, allogeneic MSCs seem to induce an immune memory represented by an increase in T cells with a memory phenotype (95).

It can be assumed that MSCs are not immune privileged, but rather that allogenic MSCs have a lower immunogenic potential as other allogeneic cell types (88). As the cytotoxic cell-induced apoptosis of MSCs was described to be essential for the MSC-mediated immunomodulation and because this effect was shown to be MHC-independent and non–antigen-specific, the allogenic component might be of secondary importance (80).

## Potential Benefit of Dead MSCs in Terms of Reduced Cancer Risk

Previous studies have suggested that MSCs could favor tumor growth *in vivo* (96, 97). It has been reported that implanted MSCs cause an earlier onset of syngeneic tumors and allow B16 melanoma cells to form tumors in allogenic mice (98). Furthermore, TNFα-activated MSCs can facilitate tumor growth and promote cancer metastasis via CXCR2^+^ neutrophil recruitment (99).

The mechanism behind MSC-induced tumor growth involves the formation of carcinoma associated fibroblasts (CAFs). Human bone marrow-derived MSCs (BM-MSCs) were shown to adopt a CAF-like phenotype with similar functional properties after prolonged exposure to tumor-conditioned medium (100, 101). CAFs and CAF-like MSCs produce growth factors, cytokines, and chemokines, and thereby provide the microvascularization and the stromal network for tumor progression (102). In a mouse model of inflammation-induced gastric cancer, at least 20% of CAFs derived from the bone marrow and developed from MSCs (103).

Furthermore, MSCs seem to have a distinct tropism for tumors as BM-MSCs were shown to accumulate in brain tumors after intracarotid injection, whereas fibroblasts and U87 glioma cells did not (20). This tropism was further elucidated in a study from 2013 that unveiled an active recruitment of MSCs to prostate cancer via prostate cancer-secreted CXCL-16. CXCL-16 binds to CXCR6 expressed by MSCs. The CXCL16/CXCR6 signaling induces the conversion of MSCs into CAFs (104). Ren et al. could show that under inflammatory conditions CAF-like MSCs stimulate tumor growth via the recruitment of monocytes and macrophages. The essential role monocytes/macrophages in MSC-mediated immunomodulation was demonstrated once more as their depletion abrogated the promotion of tumor growth by lymphoma isolated-MSCs (105).

Whilst MSCs can exhibit immunosuppressive or immune-enhancing properties depending on the presence or absence of certain inflammatory or anti-inflammatory stimuli, the tumorigenic potential of living MSCs poses a risk that cannot be neglected (26, 28, 106). As dead MSCs have no active cell metabolism, it can be assumed that they do not differentiate into CAF-like cells with the corresponding secretion of growth factors, cytokines, and chemokines. However, a clear discrimination between CAFs, CAF-like cells, and MSCs is still missing in the current literature. Furthermore, dead MSCs may still provoke changes in the tumor microenvironment due to their immunomodulatory properties. Therefore, the question whether dead or fragmented MSCs are beneficial in terms of a reduced cancer risk cannot be answered, yet.

## Conclusion

It remains a challenge to connect the dots between the various MSC-mediated immunomodulatory effects, especially as MSCs are very heterogenic and subject to significant changes upon inflammatory or anti-inflammatory stimuli. Viable MSCs might provoke more complex immunomodulatory mechanisms due to their intact secretome. However, since the discussion about a universal donor for MSC therapy has not been finally answered, the possibility to use dead MSCs should also be considered. HI-MSCs or fragmented MSCs are most likely not subject to changes in their immunomodulatory characteristics upon different environmental stimuli. Hence, their immunomodulatory effects might be more predictable, which would allow a better comparison of future study results.

## Author Contributions

AW drafted and wrote the manuscript. MD drafted and critically revised the manuscript. Both authors have approved the manuscript for publication.

### Conflict of Interest Statement

The authors declare that the research was conducted in the absence of any commercial or financial relationships that could be construed as a potential conflict of interest.
